# Definitions, measurements and prevalence of fear of childbirth: a systematic review

**DOI:** 10.1186/s12884-018-1659-7

**Published:** 2018-01-12

**Authors:** C. Nilsson, E. Hessman, H. Sjöblom, A. Dencker, E. Jangsten, M. Mollberg, H. Patel, C. Sparud-Lundin, H. Wigert, C. Begley

**Affiliations:** 1 Faculty of Caring Science, Work Life and Social Welfare, University of Borås, S-501 90 Borås, Sweden; 2 Institute of Health and Care Sciences, The Sahlgrenska Academy at University of Gothenburg, Box 457, -405 30 Gothenburg, SE Sweden; 3 Biomedical Library, Gothenburg University Library at University of Gothenburg, Box 416, -405 30 Gothenburg, SE Sweden; 4 Chair of Nursing and Midwifery, School of Nursing and Midwifery,Trinity College Dublin, 24, D’Olier St. Dublin 2, Dublin, Ireland

**Keywords:** Fear of childbirth, Systematic review, Prevalence, W-DEQ, FOBS, Request for caesarean section

## Abstract

**Background:**

Fear of Childbirth (FOC) is a common problem affecting women’s health and wellbeing, and a common reason for requesting caesarean section. The aims of this review were to summarise published research on prevalence of FOC in childbearing women and how it is defined and measured during pregnancy and postpartum, and to search for useful measures of FOC, for research as well as for clinical settings.

**Methods:**

Five bibliographic databases in March 2015 were searched for published research on FOC, using a protocol agreed a priori. The quality of selected studies was assessed independently by pairs of authors. Prevalence data, definitions and methods of measurement were extracted independently from each included study by pairs of authors. Finally, some of the country rates were combined and compared.

**Results:**

In total, 12,188 citations were identified and screened by title and abstract; 11,698 were excluded and full-text of 490 assessed for analysis. Of these, 466 were excluded leaving 24 papers included in the review, presenting prevalence of FOC from nine countries in Europe, Australia, Canada and the United States. Various definitions and measurements of FOC were used. The most frequently-used scale was the W-DEQ with various cut-off points describing moderate, severe/intense and extreme/phobic fear. Different 3-, 4-, and 5/6 point scales and visual analogue scales were also used. Country rates (as measured by seven studies using W-DEQ with ≥85 cut-off point) varied from 6.3 to 14.8%, a significant difference (chi-square = 104.44, d.f. = 6, *p* < 0.0001).

**Conclusions:**

Rates of severe FOC, measured in the same way, varied in different countries. Reasons why FOC might differ are unknown, and further research is necessary. Future studies on FOC should use the W-DEQ tool with a cut-off point of ≥85, or a more thoroughly tested version of the FOBS scale, or a three-point scale measurement of FOC using a single question as ‘Are you afraid about the birth?’ In this way, valid comparisons in research can be made. Moreover, validation of a clinical tool that is more focussed on FOC alone, and easier than the longer W-DEQ, for women to fill in and clinicians to administer, is required.

**Electronic supplementary material:**

The online version of this article (10.1186/s12884-018-1659-7) contains supplementary material, which is available to authorized users.

## Background

Being pregnant and giving birth are described as a transition phase, or an existential threshold that childbearing women have to cross [1]. Childbirth is an experience with many dimensions, multifaceted and unique for each woman, still strongly influenced by her social context [2]. Women’s expectations and experiences of pregnancy and birth are both positive and negative in nature, involving feelings of joy and faith but also worries, anxiety and fears. Despite the fact that maternity care in high income countries is safe, fear of childbirth is a common problem affecting women’s health and wellbeing before and during pregnancy, as well as after childbirth. Fear of childbirth has consequences for women’s relationships with their baby, partner and family [3], and often leads to requests for caesarean section (CS) by women striving for control in an exposed situation [4–7].

During the last few decades there has been a growing research interest in women’s fear of childbirth. For some women, the fear only relates to childbirth, but for others fear occurs in relation to other types of anxiety also [3, 8]. Fear of parturition is not new, and was described by the French psychiatrist Louis Victor Marcé (1858) [9]. The term ‘fear of childbirth’ (FOC) was characterised in 1981 in a population of Swedish pregnant women, defined as: “a strong anxiety which had impaired their [the women’s] daily functioning and wellbeing” [10, p. 265]. In addition, a more moderate fear was described as a significant anxiety, which did not interfere with the women’s daily life [10]. Later, during the 1990s, studies from Finland defined FOC as a health issue for a pregnant woman related to an anxiety disorder or a phobic fear including physical complications, nightmares and concentration problems, as well as demands for caesarean section [11]. The term ‘clinical FOC’ describes a “disabling fear that interferes with occupational and domestic functioning, as well as social activities and relationships”, and in some cases even reaches the classification for a specific phobia according to the DSM IV [3, p. 141]. The label “tokophobia” is also used [12, 13], characterised as an “unreasoning dread of childbirth” in women, a “specific and harrowing condition” [12, p. 83] including a “pathological dread” and “avoidance of childbirth” [13, p. 506]. Moreover, FOC is strongly related to the increasing caesarean section (CS) rates in Western countries, as being a common cause for women requesting a surgical birth [14, 15].

In early studies, the prevalence of FOC for pregnant women in Scandinavia was reported as 20%, with approximately 5–10% women experiencing intense fear [10]. The prevalence in Europe seems to vary between countries, from 1.9–14% [16], while Australia indicates higher rates of around 30% [17], leaving questions on possible cultural differences in FOC, or differing definitions. However, the variations in prevalence can also depend on the measures used; these can vary from fear being self-defined by women, or self-reported via different questionnaires, or estimated through measurement of physiological indices such as stress hormones in childbirth [3, 18, 19].

For women lacking experience of childbirth (so called primary tokophobia or FOC), their fears may date from adolescence or early adulthood, where experiences of others’ fearful responses to childbirth or a history of anxiety disorders could be important [8, 12, 13]. Secondary tokophobia or FOC is related to the event of birth, and is usually linked to fears developed after a previous negative or traumatic experience of childbirth, sometimes related to posttraumatic stress disorder (PTSD) [3, 8, 11–13]. Tokophobia is also described as a symptom of prenatal depression [12, 13]. However, the research on FOC has been criticised for constructing women’s fear as a medical category, having too pathological an approach that searches for errors in women, instead of examining possible causes of women’s fear within maternity care itself [20, 21].

To summarise, the research field is extensive, complex, and difficult to survey without any consensus on definitions. The concept “fear of childbirth” seems to be used as a broad label for all kinds of anxiety and fears that women experience in relation to pregnancy and childbirth. Relevant questions are: How common is FOC? Are there any cross cultural differences? Which measures are pertinent? What is FOC? There is a need for systematic reviews on FOC to be able to direct future research on developing optimal care and effective treatment for women fearing childbirth and to identify factors that reduce, as well as increase, women’s fears. Therefore, as a first step, we conducted a systematic review of all studies demonstrating a prevalence of FOC. The aims of this review were to identify the prevalence of FOC in childbearing women and how it is defined and measured during pregnancy and postpartum, and to search for useful measures of FOC, for research as well as for clinical settings.

## Methods

### Inclusion criteria

#### Types of participants

Participants were childbearing women (defined as the period covering pregnancy, labour and birth, and the first year postpartum).

#### Types of studies

Surveys, cross-sectional studies, experimental and quasi-experimental studies (where the control group could provide data), observational studies, systematic reviews, and meta-analyses, were eligible for inclusion.

#### Types of outcome

The primary outcome was prevalence of fear of childbirth, where this was defined clearly by study authors. Papers measuring fear during labour were excluded. Many studies used the same population (i.e., a number of PhD students accessed the same population at the same time and conducted different studies, but reported the same prevalence). We only included studies that were the first to report prevalence in a population and at similar time points. Studies reporting the same prevalence, on the same population or on a subsample (less representative, were excluded.

### Search and selection strategy

A search strategy was developed and reviewed for accuracy, by one member not involved in its development (CS-L), using the Peer Review of Electronic Search Strategies (PRESS) criteria [22]. No restrictions were applied to years searched, but papers included were limited to English and Swedish publications only. We searched electronic bibliographic databases of The Cochrane Library, PubMed, Scopus, PsycINFO and CINAHL from their inception dates, in March 2015, using the agreed search strategy as described in Additional file 1.

### Selection of studies

Studies were selected for inclusion from the papers identified, by team members working in pairs, using the above criteria. Any disagreements were resolved by a third member.

### Quality assessment of included studies

The Effective Public Health Practice Project (EPHPP) quality assessment tool [23] was chosen to assess methodological quality of all studies included. Components of study design and methods assessed by this tool include selection and allocation bias, confounding, blinding, methods of data collection, withdrawals/drop-outs and analysis and intervention integrity. As some dimensions of the EPHPP (e.g. the sections on confounding and intervention integrity) are not relevant for reviews of non-intervention studies, the tool did have some shortcomings. For example, following discussion, it was agreed by the team to rate all cross-sectional studies (single cohort) as moderate, to avoid studies of otherwise good quality being excluded. Despite these problems, the tool was useful, especially for identifying ‘Weak’ experimental studies and excluding them. An overall quality rating was assigned to each study following assessment, of Strong (where no weak ratings were assigned), Moderate (one weak rating) or Weak (two or more weak ratings). An a priori decision was made that studies receiving a ‘Weak’ global rating score would be excluded from analysis. Team members in pairs assessed the quality of included studies. Any disagreements were discussed and resolved by consensus, or by a third member of the review team if necessary. In addition, as one of the team-members was co-author in some of the studies, those were assessed by other members of the review team.

### Data extraction and analysis

Using a pre-designed data extraction form, data on prevalence, definitions and measurements were extracted independently by each member of the four review teams and checked for accuracy by the other reviewer.

Due to the differing types of studies, it was seldom possible to combine results into a meta-analysis. A narrative synthesis was provided instead.

## Results

### Results of search and selection strategy

**Fig. 1:**
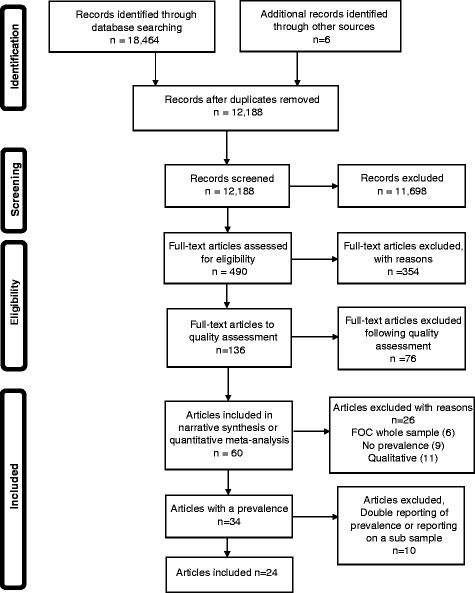
Prisma flow chart Single file. Prisma flow-diagram of the search and selection process

In total, 18,464 citations were identified using the search strategy designed. After removing duplicates, 12,188 unique citations were screened by title and abstract and 11,698 excluded. Full-text papers of the remaining 490 citations were read and 354 of these were subsequently excluded, leaving 136 for inclusion (Fig. 1).

### Methodological quality of included studies

The 136 papers were assessed and 76 were rated as “Weak”, and therefore excluded. The main reasons for “Weak” ratings were that the samples were unlikely to be representative of the population and the data collection tools were either not tested for validity, or there was insufficient information on their testing. This resulted in 60 papers meeting the inclusion criteria for this review (Fig. 1). At data extraction stage, however, it was clear that, for nine of the papers, no prevalence data could be identified [24–32], for six others, the whole sample of women had FOC [33–38], 11 papers were qualitative [39–49], and 10 papers had double reporting or reported the prevalence of a subsample of a population already included in the review [50–59]; these were excluded leaving a total of 24 papers for inclusion, based on data from 23 study populations (two papers [60, 61] presented FOC during pregnancy and postpartum, based on the same population) (Fig. 1 and Table 1). Thirteen of these had been rated as methodologically “Strong” and 11 were rated as “Moderate” (Table 1).

**Table 1:** Characteristics of included studies on Fear of Childbirth

First author, year of publication [reference number]	EP-HPP rating	Aim of study	Study design	Population	Sample size (response rate, %)	Country
Adams 2012 [80]	S	To assess the association between FOC and duration of labour	Prospective cohort study, using postal survey at 32 weeks	All women scheduled to give birth at a University hospital Nov 2008-April 2010	2206 (63.0)	Norway
Elvander 2013 [63]	M	To estimate the effects of different levels of fear of birth and mode of delivery on birth experience 1 month after birth	Prospective study, using telephone interviews in the third trimester of pregnancy and 1 month after birth	Nulliparous English and Spanish-speaking women aged 18–35, with a single fetus, who birthed >34 weeks’, recruited in a variety of ways, in 2009–2011	3005 (NA) Number eligible not mentioned	USA
Eriksson 2005 [71]	S	To investigate and compare experiential factors associated with childbirth-related fear in women (and men)	Cross-sectional observational study, using postal survey 14–26 months postnatal. FOC was assessed retrospectively	All women who had a baby in a University hospital, March 1997–March 1998	410 (73.5)	Sweden
Fabian 2004 [73]	M	To investigate the attendance rate at childbirth and parenthood education classes // and describe the characteristics of women who did not attend	Cohort study using a postal questionnaire in early pregnancy and at 2 months postpartum	All women attending 97% of all antenatal clinics in Sweden for 1 week in May and September 1999, and January 2000	2546 (55.0)	Sweden
Fenwick 2009 [77]	M	To investigate levels of pre- and postpartum of childbirth fear in a cohort of childbearing women and explore the relationship to birth outcomes	A prospective correlation design using postal surveys at 36 weeks’ gestation and 6 weeks’ postpartum	All women (English-speaking with a single healthy fetus) attending antenatal clinic at a tertiary hospital**,** September 2005–March 2006	401 (43.0)	Australia
Geissbuehler 2002 [69]	M	To examine the intensity and type of childbirth fears among pregnant women in the 2nd-3rd trimester // to consider whether birth preparation influences childbirth anxiety	Cross-sectional, self-administered survey in 24th–28th weeks	All women booked to give birth in a large hospital, November 1991–October 1999	8528 (79.1)	Switzerland
Haines 2011 [67]	S	To examine the prevalence of childbirth-related fear (CBRF) in two rural populations (Sweden and Australia) and to pilot a short easy-to-administer tool	Cross-sectional study, using postal survey at 18 weeks gestation	Women undergoing routine ultrasound at 17–19 weeks at a regional Swedish hospital during 2007 and women booked booked at 18–20 weeks in an Australian regional hospital, year not described	509 (NA) (Sweden: *n* = 386 and Australia: *n* = 123) Number eligible not mentioned	Sweden and Australia
Hall 2009 [85]	S	To explore women’s levels of childbirth fear, sleep deprivation, anxiety, and fatigue and their relationships during the 3rd trimester of pregnancy	Cross-sectional using surveys at term in a convenience sample at 35–39 weeks	English speaking low-risk women at 35–39 weeks gestation from May 2005–July 2007	650 (NA) Number eligible not mentioned	Canada
Heimstad 2006 [78]	M	To estimate prevalence of FOC in a defined area in Norway and to study the possible relationship between FOC and psychosocial background, degree of anxiety and abuse	Cross-sectional study, using postal questionnaires at 18–20 weeks	All pregnant women scheduled for a routine ultrasound at a University Hospital, June 2001–August 2002	1452 (54.2)	Norway
Hildingsson 2010 [60]	S	To describe and study background characteristics, feelings and support in relation to thoughts about childbirth in mid-pregnancy	Cross-sectional study, using self-administered surveys at 17–19 weeks	All pregnant women who had a routine ultrasound at three hospitals in one region, year 2007	1212 (51.4)	Sweden
Jespersen 2014 [81]	S	To assess the association between FOC and emergency caesarean section	Prospective cohort study using 2 questionnaires; at 37 weeks gestation and at admission to labour ward	All nulliparous, pregnant low risk women in spontaneous labour at 4 major university hospitals, 3 country hospitals and 2 local district departments, May 2004–July 2005	2598 (71.1)	Denmark
Jokić-Begić 2014 [82]	S	To examine the role of demographic variables, expected pain level, trait anxiety and anxiety sensitivity in FOC among nulliparous and multi-parous women in the last trimester of pregnancy	Cross-sectional/single cohort study using one questionnaire in 8th–9th months of pregnancy	Pregnant women attending at a perinatal clinic at one University hospital in Zagreb, Jan-May 2012	200 (67.6)	Croatia
Laursen 2008 [64]	M	To describe the association between FOC and social, demographics and psychological factors in healthy nulliparous women with uncomplicated pregnancies	Population-based prospective cohort study (pre-post design), using telephone interviews at 16 and 32 weeks	All nulliparous women with uncomplicated pregnancies, 1997–2003	30,480 (approx. 30%)	Denmark
Lowe 2000 [74]	M	To test whether the theoretically predicted inverse relationship between childbirth self-efficacy and fear exists, and to characterize specific personality attributes associated with women who express low or high FOC	Cross-sectional cohort study (secondary analysis), using the Childbirth Attitudes Questionnaire distributed in the third trimester and returned by post	Nulliparous women enrolled in childbirth education classes, year for data collection not described	280 (NA) Number eligible not mentioned	USA
Lukasse 2014 [62]	M	To assess the prevalence of severe FOC and investigate the association between severe FOC and selected background variables	Cohort study, using a self-completed survey in pregnancy at varying times	Pregnant women attending antenatal care in 6 countries between March 2008–August 2010	6870; 828 (B) 585 (I), 1252 (D) 896 (E) 2351 (N) 958 (S) (NA)	Belgium, Norway, Iceland, Denmark Estonia Sweden
Nieminen 2009 [79]	M	Investigate the prevalence of intense FOC, association between the level of FOC and gestational age, the risk factors for intense FOC in primi and multiparous, and risk factors associated with preference for caesarean section	Cross-sectional, using a self-completed survey in pregnancy	All Swedish speaking primi and multiparous women in four districts, September–October 2006	1635 (98.3)	Sweden
Nilsson 2012 [61]	S	To explore FOC during pregnancy and 1 year after birth and its association with birth experience and mode of birth	A population based prospective longitudinal survey (pre and post design), using postal surveys in mid and late pregnancy, 2 months and 1 year postpartum	All pregnant women who had a routine ultrasound at three hospitals in one region, year 2007 (Same population as in Hildingsson 2010)	763 (86)	Sweden
Poikkeus 2006 [75]	M	To compare the prevalence and predictors of severe FOC and pregnancy related anxiety in groups of assisted reproduction treatment (ART) and spontaneously conceiving women with singleton pregnancies	Prospective longitudinal study with cohort (ART) and matched control (consecutive enrolment) groups, using a self-completed survey at 20 weeks	ART group = 367, and consecutive controls = 379, year 1999	746 (86.6)	Finland
Rouhe 2015 [65]	S	To assess effects of psycho-education versus conventional care during pregnancy in women with FOC	All nulliparous women at time of routine ultrasound at 11–13 weeks. 371 women with severe FOC participated in an RCT using psycho-education as relaxation (6 sessions during pregnancy, one postnatal) and conventional care by community nurses Questionnaires completed twice during pregnancy and/or 3 months postpartum	4575 screened by W-DEQ for severe FOC during routine ultra-sonography at 11–13 weeks. Those with scores >100 were included in the trial, in October 2007–August 2009	4575 (NA) Number eligible not mentioned	Finland
Räisänen 2014 [66]	S	To identify risk factors for FOC and evaluate relation between FOC and adverse perinatal outcomes	Cohort register study, using The Finnish Medical Birth Register, with FOC defined according to ICD – code 099.80	All singleton births during 1997–2010	788,317 (100?)	Finland
Söderqvist 2004 [83]	S	To investigate association between traumatic stress symptoms and FOC in late pregnancy	Cohort using self-completed survey at week 32	Consecutive recruitment of pregnant women visiting hospital in Kalmar and Linköping, in 1997	951 (48.2)	Sweden
Ternström 2014 [72]	S	To investigate the prevalence of childbirth-related fear in early pregnancy among Swedish and foreign-born women living in Sweden	Cross-sectional study of a total population attending ultrasound screening, using a self-completed questionnaire at 17–20 weeks	University hospital, 615 women screened during routine ultra-sonography, and asked to participate, year not described	606 (96.2)	Sweden
Waldenström 2006 [70]	M	To investigate the prevalence of FOC in a nationwide sample and its association with subsequent rates of CS and overall experience of childbirth	A longitudinal national cohort study, using postal survey at 16 weeks gestation and 2 months postpartum	All pregnant women invited to participate at 16th week gestation and at 2 months postpartum, 3 weeks: May and September 1999 and January 2000	2662 (97.0)	Sweden
Zar 2002 [84]	S	To investigate the prevalence of extreme FOC and anxiety disorders in late pregnancy	Prospective study using postal survey at week 28–30 and interview at 32 weeks	Pregnant women from a country hospital were invited to participate, during 8 months, year not described	506 (82.5)	Sweden

*M* Moderate, *S* Strong, *EPHPP* Effective Public Health Practice Project, *FOC* Fear Of Childbirth, *CS* Caesarean Section

### Description of studies

The studies had been conducted in twelve countries, with Sweden emerging as the country with the most research into FOC (Table 1). Ten papers (reporting on nine studies) were from Sweden (one of which also included a cohort of women from Australia), two from Norway, four from Finland, two from Denmark, two from the United States of America, and one each from Canada, Australia, Switzerland, and Croatia. One study had included data from six countries: Belgium, Norway, Iceland, Denmark, Estonia, and Sweden [62]. The majority (*n* = 20) had collected data using either postal or self-completed and personally returned surveys, one in the post-natal period, 16 in the antenatal period and six at both time periods. Two studies used telephone interviews, one in the third trimester of pregnancy and 1 month after birth [63] and the second in early and late pregnancy [64]. One study used a randomised controlled trial methodology and we used the data from the population screened by W-DEQ to identify FOC in early pregnancy, prior to trial entry [65]. One study [66] used a retrospective analysis of a national database to gather data on FOC, defined according to ICD code 099.80 (Table 1). Sample sizes ranged from 200 to 788,317.

Eight papers were published based on studies using the same population, with surveys administered at four time-points, and care has been taken to ensure that rates have not been double-counted. Of these eight papers we only included three [60, 61, 67]; the others were excluded due to repeated prevalence reporting or because they reported on a subsample of a previously reported population [50–54]. Hildingsson et al. 2010 [60] included 1212 women and presented the results for FOC in mid-pregnancy, and Nilsson et al. [61] used the same sample, but presented the results for FOC in women 1 year after birth (*n* = 763). Haines et al. [67] took a sub-set of 386 women from the full population, who had attended one regional hospital, and recruited 122 Australian women also. As a VAS was used for the response format, these results are presented separately and are not merged with any other.

### Definitions and measurements of FOC used by included studies

Eleven papers used the W-DEQ questionnaire, an instrument specifically designed to measure fear of labour and birth (Table 2). It consists of a 33-item questionnaire, and can be scored from 0 to 165 [68]. Various different cut-off points are used to define ‘severe fear of birth,’ from scores of ‘66 or greater’ to ‘greater than 100’, which makes comparison of prevalence difficult.

**Table 2:** Measurement tools used, prevalence, and parity in Fear of Childbirth studies

First author, year of publication, [reference number]	Measurement tools used, in descending order of cut-off point	Level of FOC	Time point of FOC	Prevalence, % (95% CI)	Number	Parity group^a^
	W-DEQ					
Rouhe 2015 [65]	W-DEQ, with score ≥ 100	Very severe FOC	Early pregnancy	8.1 (7.3–8.9)	371/4575	1
Heimstad 2006 [78]	W-DEQ score > 100 W-DEQ > 95	‘Serious FOC; of clinical importance’	Mid pregnancy	5.5 (4.3–6.8) 7.3 (5.9–8.8)	72/1321 96/1321	2
Nieminen 2009 [79]	W-DEQ score ≥ 100 W-DEQ score ≥ 85	Very intense FOC Intense FOC	Pregnancy (various times)	5.7 (4.6–6.9) 15.6 (13.8–17.4)	93/1635 254/1635	2
Adams 2012 [80]	W-DEQ score ≥ 85	‘High fear’	At 32 weeks	7.5 (6.4–8.7)	165/2206	2
Jespersen 2014 [81]	W-DEQ score ≥ 85	Severe FOC	At 37 weeks	9.0 (7.8–10.2)	207/2310	1
Jokić-Begić 2014 [82]	W-DEQ score ≥ 85	FOC	Late pregnancy	11.5 (7.4–16.8)	23/200	2
Lukasse 2014 [62]	W-DEQ score ≥ 85	Severe FOC	In pregnancy (various times)	11.2 (total population in six countries) ^b^(10.5–12.0) Belgium: 6.3 (4.7–8.2) Iceland: 8.4 (6.3–10.9) Denmark: 9.2 (7.6–10.9) Norway: 11.8 (10.5–13.2) Sweden: 14.8 (12.6–17.2) Estonia: 14.8 (12.6–17.3)	Total: 769/6870 B: 52/828 I: 49/585 D: 115/1252 N: 278/2351 S: 142/958 E: 133/896	2
Söderqvist 2004 [83]	W-DEQ score ≥ 85	Severe FOC	Late pregnancy	13.5 (11.4–15.8)	127/942	2
Zar 2002 [84]	W-DEQ score ≥ 85	Severe FOC	Late pregnancy	11.1 (8.5–14.1)	56/506	2
Fenwick 2009 [77]	W-DEQ score ≥ 71	‘High level of fear’	Late pregnancy 6 weeks postpartum	26.2 (21.9–30.8) 22.4 (17.3–28.1)	105/401 55/246	2
Hall 2009 [85]	W-DEQ score ≥ 66	‘High fear’	Late pregnancy	24.9 (21.6–28.4)	162/650	2
	Fear of Birth scale (FOBS)					
Haines 2011 [67]	Fear of Birth Scale (FOBS) score > 50.	Elevated level of FOC	Mid-pregnancy	Sweden: 31.1 (26.5–36.0) Australia: 29.5 (21.6–38.4)	119/383 36/122	2
Ternström 2014 [72]	Fear of Birth Scale (FOBS) score > =60.	Childbirth related fear (CBRF)	Mid-pregnancy	22.1 (18.9–25.6)	134/606	2
	Various scales to measure FOC					
Elvander 2013 [63]	First Baby Study Birth Anticipation Scale score 21–30	High fear	Late pregnancy	20.3 (18.9–21.8)	611/3005	1
Lowe 2000 [74]	Childbirth Attitudes Questionnaire, 1 Standard Deviation above mean	High fear	Late pregnancy	19.3 (14.8–24.4)	54/280	1
Poikkeus 2006 [75]	Revised Fear-of-Childbirth questionnaire, total score ≥ 6	Severe fear	Mid pregnancy	11.0 (8.8–13.5)	82/746	2
	Single item question – 3 point Likert response scale					
Geissbuehler 2002 [69]	‘Are you anxious or afraid about the birth?’, answer by ‘Yes, very afraid’	FOC	Late pregnancy	5.3 (4.8–5.8)	450/8528	2
Laursen 2008 [64]	‘Are you anxious about the course of the upcoming delivery?’ answered with ‘A lot‘	FOC	Early pregnancy Late pregnancy	7.6 (7.3–7.9) 7.4 (7.1–7.7)	2308/30,480 2245/30,480	1
	Single item question – 4 point Likert response scale					
Hildingsson 2010 [60]	’How do you feel when thinking about labour and birth?’ answered with: ‘A lot/very much’	Childbirth related fear	Mid pregnancy	14.0 (12.1–16.1)	170/1212	2
Nilsson 2012 [61]	’To what extent do you experience worries and fear?’ answered with: ‘A great deal/very much’ ^c^	FOC	1 year after childbirth	15.1 (12.6–17.9)	115/761	2
	Single item question – 5/6 point Likert response scale					
Eriksson 2005 [71]	A statement ‘Childbirth-related fear influences my daily life in a negative sense’ with a 6-point Likert scale	Intense fear	Assessed retrospectively	22.9 (18.9–27.3)	94/410	2
Waldenström 2006 [70]	A question: ‘How do you feel when thinking about labour and birth?‘answered by ‘Very negative‘	Childbirth related fear	Early pregnancy	3.6 (3.0–4.4)	97/2662	2
	Miscellaneous					
Fabian 2004 [73]	Yes to a question on whether they had attended/needed to attend a clinic for counseling because of FOC	FOC	Early pregnancy	15.4 (14.0–16.9)	385/2503	2
Räisänen 2014 [66]	Register study. FOC defined according to ICD – code 099.80	FOC	Pregnancy	3.7 (3.6–3.7)	28,960/ 788,317	2

*VAS* Visual Analogue Scale, *FOC* Fear Of Childbirth

^a^Parity group 1 = nulli/primiparous women only, 2 = both primi- and multiparous women

^b^Significant difference in country rates (chi-square = 55.5, d.f. = 5, *p* < 0.0001)

^c^Same study population as in Hildingsson 2010

The authors of two papers [64, 69] used a 3-point scale to measure fear/anxiety about birth, in answer to similar questions relating to FOC (Table 2). The three response options were variously expressed as: “no, I am not afraid/not at all;” “yes, I am a bit afraid/yes, a little;” “yes, I am very afraid/yes, a lot,” respectively, which were deemed suitable to merge (Table 2). Their definition of severe FOC was thus “yes, I am very afraid/yes, a lot.” Laursen et al. [64] measured FOC in both early and late pregnancy, but as the other authors, Geissbuehler and Eberhard [69], measured FOC in late pregnancy only, a combination of the late pregnancy rates was made.

A question with a four-point response scale was used in a study of a Swedish population, reported by Hildingsson et al. [60], and Nilsson et al. [61], with all their data coming from the same cohort of women and using similar questions relating to FOC; “to what extent do you experience worries and fear?”, With the addition of “when thinking of coming births” in the questionnaire 1 year after birth (Table 2). The authors dichotomised the scale into ‘no fear’ and ‘fear’. ‘No fear’ was described variously as ‘not at all + very little’ and ‘not at all + somewhat’, and ‘fear’ was described as ‘a lot + very much’ and ‘a great deal + very much’ (Table 2).

The remaining papers used a heterogeneous mix of various scales for measurement, none of which could be combined. Elvander et al. [63] graded fear on a 5-point scale, where women answered 6 questions on feeling: ‘nervous,’ ‘worried,’ ‘fearful,’ ‘relaxed,’ ‘terrified,’ and ‘calm’, in relation to their impending birth. Scores were divided into 3 categories: 6–13 (low fear), 14–20 (intermediate fear) and 21–30 (high fear, their definition of FOC). Waldenström et al. [70] also assessed FOC on a 5-point rating scale using the single question: “How do you feel when thinking about labour and birth?” Women ticking the “very negative” response alternative were defined as having childbirth related fear. Eriksson et al. [71] graded fear on a 6-point scale, from “no fear at all” to “very high fear.” Intense fear was defined as four or above and agreement with the statement that “childbirth-related fear influences your daily life in a negative sense” (Table 2).

Two studies [67, 72] used the Fear of Birth (FOBS) visual analogue scale but with different cut-off points (>50 and ≥60), so results could not be merged. The FOBS scale consists of one question – “How do you feel right now about the approaching birth?”- graded by marking two 100 mm visual analogue scales, which are anchored with the words “calm/worried” and “no fear/strong fear” [67].

Fabian et al. [73] asked women to answer one question on whether they had attended, or felt they needed to attend, a clinic for counseling in relation to FOC, to which they answered ‘yes’ or ‘no’. Lowe et al. [74] used a 15 item Childbirth Attitudes Questionnaire (high fear = 1 Standard Deviation above mean) and Poikkeus et al. [75] used a revised version of the Fear-of-Childbirth questionnaire, developed by Saisto et al. [76], and pregnancy anxiety scale. Total scores equal to or higher than the 90th percentile in the revised Fear-of-Childbirth questionnaire (total scores ≥6 and pregnancy anxiety scale, total scores ≥30) were considered “severe fear” and “severe pregnancy related anxiety” (Table 2). None of these results could be combined.

The majority of studies took measurements at only one time point or at varying times throughout pregnancy without comparing rates from different times. Laursen et al. [64] found rates in early pregnancy to be 2308 out of 30,480 (7.6%), similar to rates in late pregnancy, of 2245 out of 30,480 (7.4%). Postpartum rates of FOC were measured in only two studies [61, 77]. Due to differing measurement times and tools, no comparisons could be made but postpartum rates did not seem to differ greatly from those in the antenatal period (Table 2).

### Prevalence

The prevalence of FOC varied, which may be due, in part, to the differing measurement scales. The most common scale was the W-DEQ, used in 11 studies, with four different cut-off points (some papers used more than one point). Three studies [65, 78, 79] used a cut-off point of greater than or equal to 100, with rates varying from 5.5 to 8.1%, giving an average ‘very severe’ FOC rate of 7.1% (536 out of 7531). A cut-off of greater than or equal to 85 in seven studies [62, 79–84] two cut-off levels gave FOC rates of 7.5% (165 out of 2206) to 15.5% (254 out of 1635), giving an average ‘severe’ rate of FOC of 11.1% (1545 out of 14,163) (Table 2). The final two studies using W-DEQ had cut-off points of greater than or equal to 66 [85] or 71 [77], which gave rates of ‘moderate’ FOC of 24.9 - 26.2% (Table 2).

The two studies using a 3-point scale [64, 69] had merged “extreme fear” FOC rates of 7.0% (2707 out of 38,801). The data collection using the dichotomised 4-point scale had rates (based on the same population) of 14% [60], in mid pregnancy, and 15.1% 1 year after birth [61] (Table 2).

Rates for the studies using 5- and 6-point scales [70, 71] varied considerably from 3.6 to 22.9%, and the 5-point scale was based on the question “How do you feel when thinking about labour and birth?” with the response “very negative” deemed to equate to FOC [70], which may not be accurate. Rates for all other prospective studies varied from 11 to 31.1%; the register study based on data from medical records [66] showed a rate of 28,960 out of 788,317 (3.7%) (Table 2).

### Comparison of country rates

Rates of severe FOC in each country (as measured by seven studies using W-DEQ with ≥85 cut-off point) were combined, the average taken for each country, and then compared. The average rates varied from 6.3% in Belgium to 14.8% in Estonia, a significant difference in the seven countries (chi-square = 104.44, d.f. = 6, *p* < 0.0001) (Table 3).

**Table 3:** Prevalence of FOC in different countries, measured by W-DEQ (cut-off ≥85)

First author, year of publication	Prevalence	Country total	Average country prevalence
Lukasse 2014	142 out of 958 (14.8%)	Sweden	579 out of 4041 (14.3%)
Nieminen 2009	254 out of 1635 (15.5%)^a^		
Söderqvist 2004	127 out of 942 (13.5%)		
Zar 2002	56 out of 506 (11%)		
Adams 2012	165 out of 2206 (7.5%)	Norway	443 out of 4557 (9.7%)
Lukasse 2014	278 out of 2351 (11.8%)		
Jespersen 2014	207 out of 2310 (9.0%)	Denmark	322 out of 3562 (9.0%)
Lukasse 2014	115 out of 1252 (9.2%)		
Jokić-Begić 2014		Croatia	23 out of 200 (11.5%)
Lukasse 2014		Belgium	Belgium: 52 out of 828 (6.3%)
		Iceland	Iceland: 49 out of 585 (8.4%)
		Estonia	Estonia: 133 out of 896 (14.8%)

^a^Calculation performed by review authors

## Discussion

W-DEQ gave an average ‘very severe’ (greater than or equal to 100) FOC rate of 7.1%, severe (greater than or equal to 85) rate of 11.1%, and a ‘moderate’ rate of 25.3 - 26.2% (greater than or equal to 66 or 71). Merged rates from two studies [64, 69] using a question with a 3-point response scale and similar questions on fear (Table 2) had an average “extreme fear” rate in early pregnancy of 7%, very similar to the “very severe” rates measured by W-DEQ. The study using a similar question with a dichotomised 4-point Likert response scale (Table 2) had a rate in early pregnancy of 14.0% for fear or strong fear of childbirth [60].

Once W-DEQ cut-off points went below 85, or other scales had more than three (or four) points, the rates increased and also varied considerably from the average seen at the cut-off ≥85. This might indicate that using the W-DEQ tool with a cut-off point of ≥85, or a measurement of FOC using a single question such as ‘Are you afraid about the birth?’, with three responses such as ‘no, I am not afraid; yes, I am a bit afraid; yes, I am very afraid’ [69] would measure FOC equally but that needs further testing.

Rouhe et al. [86] have previously compared FOC levels in 1348 women using the 33-item W-DEQ scale (with a cut-off point of ≥85) with a one-item VAS scale, and a cut-off point of 5. Although there was some correlation they found it not to be as accurate as the W-DEQ, but pronounced it suitable for initial screening. Haines et al. [87] also compared the W-DEQ scale (using a cut-off point of ≥85) with the Fear of Birth scale (FOBS), a two-item VAS scale, involving 1410 women. Results showed a strong correlation. However, the FOBS’s cut-off point was 54, approximately equivalent to the cut-off of 5 used in their work examined here [67], which resulted in quite high FOC rates.

Using the question with a four-point Likert response scale appeared to over-estimate FOC rates, especially when results were dichotomised so that ‘extreme fear’ was merged with more moderate fear. Dichotomising results before analysis appears to be common, but ultimately does not produce useful results if a true measure of severe FOC is the main aim.

Rates of severe FOC, measured in the same way, varied in different countries from 6.3 to 14.8%. These results are new as, although one study in this review involved measuring FOC in six countries, no paper has analysed data from all seven countries where FOC has been measured. Reasons why FOC might differ in different countries are unknown, and further research in this field is required. One reason may be poor translation, or insufficient testing of the translated version of W-DEQ, both problems highlighted by previous authors [77, 88]. Another possible explanation is that some factors may remain unidentified when measuring with W-DEQ. For instance most scales measuring fear of childbirth do not consider important dimensions such as fear of abandonment by staff during birth [89], fear of medical interventions, loss of autonomy and control, as well as fear of mistreatment and obstetrical violence [90]. In addition, the W-DEQ scale assesses a range of emotions about labour and birth, where fear is only one emotion among many others. However, despite its shortcomings, the W-DEQ has been highlighted in a recent systematic review on validated instruments used for measuring women’s childbirth experiences as, currently, the best, most used and validated tool to measure FOC [91]. Culture-specific aspects in relation to fear of childbirth have been recognised in medicalised birth cultures, where young adults prefer CS over vaginal birth, and negative impressions of birth through visual media can be an important factor for generating fear [92, 93]. Moreover, traditions surrounding birth, women’s rights, how antenatal and maternity care is organised, CS rates, and which professions (midwives, GPs, obstetricians) are involved in pregnant and childbearing women’s care, could all influence women’s fear of childbirth.

As FOC has been shown in a large systematic review and meta-analysis to be strongly associated with post-traumatic stress disorder [94], simple and early diagnosis and intervention for women with severe FOC is recommended. Antenatal education, a relatively cheap and cost-effective intervention has been shown to decrease fear of childbirth [95, 96], as has cognitive behavioural therapy, although the sample size was small [97]. We therefore also examined the published tools to see which might be most useful in the clinical area. As shorter tools have greater clinical utility than the W-DEQ, a comparison between W-DEQ and a measurement of FOC using a single question with three or four responses (that are not dichotomised before analysis) would be useful in making decisions as to how best to measure FOC swiftly and accurately in the clinical location. A comparison between W-DEQ and the FOBS scale, possibly after further testing using a higher cut-off point, would also be very useful to the research and maternity care communities.

## Conclusions

Rates of severe FOC, measured in the same way, varied in different countries. Reasons why FOC might differ are unknown, and further research is necessary. Using the W-DEQ tool with a cut-off point of ≥85, or a measurement tool using a single question with three responses, are consistent in measuring levels of severe FOC. Research comparing W-DEQ and a measurement tool using a single question with three responses, or four responses that are not dichotomised before analysis, would be useful to both the research and clinical communities. Similarly, continued and further testing of the FOBS scale, especially using a higher cut-off point to separate out “severe” FOC from more moderate levels, could prove beneficial to clinicians.

Validation of a simpler tool like the FOBS or a single question is required as there is a need for a tool that is easy and quick for women to fill in, as not all clinicians have time to administer the longer W-DEQ. Newly-developed, untested scales cannot easily be compared with other studies, and should not be used in clinical practice without further testing.

We recommend that future studies on FOC should use either the W-DEQ tool with a cut-off point of ≥85, or a more thoroughly tested version of either the FOBS scale with a higher cut-off point, or a single question such as ‘Are you afraid about the birth?’ with a three- or un-dichotimised four-point Likert response scale. In this way, valid comparisons can be made between countries and other studies.

Further research is also needed into reasons why FOC might differ in different countries and whether care for women with FOC needs to be made culturally specific. Measurement of FOC needs to include aspects such as fear of abandonment by staff during birth, fear of medical interventions, loss of autonomy and control, as well as fear of mistreatment and obstetrical violence. This more focused research agenda to guide future studies will result in more meaningful results that can be used to improve care provided for all women with fear of childbirth.

## Additional file

Additional file 1: Search strategy. (DOCX 96 kb)

## Abbreviations

CS: Caesarean section
FOBS: Fear of birth scale
FOC: Fear of childbirth
VAS: Visual analogue scale
W-DEQ: The Wijma delivery expectancy/ experience questionnaire
