# Supplementary material for: Definitions, measurements and prevalence of fear of childbirth: a systematic review
Source: BMC Pregnancy Childbirth. 2018 Jan 12;18:28. doi: 10.1186/s12884-018-1659-7 (PMC5766978; doi:10.1186/s12884-018-1659-7)
Supplement: Additional file 1: — Search strategy. (DOCX 96 kb) [file 12884_2018_1659_MOESM1_ESM.docx]

**Additional file 1**

**Search strategy, March 23-24 2015**

**PubMed**((((fear OR anxiety OR "birth trauma" OR PTSD OR stress disorders))) AND ((birth OR childbirth OR "post partum" OR postpartum OR "post natal" OR postnatal OR puerperium OR antenatal OR prenatal OR perinatal OR parturition OR delivery, obstetric OR cesarean section OR Extraction, Obstetrical OR labor, induced))) AND (("Randomized Controlled Trial"[Publication Type] OR "Observational Study"[Publication Type] OR "Clinical Trial"[Publication Type] OR "Randomized Controlled Trials as Topic"[Mesh Terms] OR "Placebos"[Mesh Terms] OR (random* AND trial*[tiab]) OR "randomized"[tiab] OR "randomly"[tiab] OR placebo* OR "Review" OR "meta-analysis" OR questionnaire OR interview OR survey OR cohort study OR focus group))

LIMITS: language: English, Swedish

**CINAHL**(birth OR childbirth OR "post partum" OR postpartum OR "post natal" OR postnatal OR puerperium OR antenatal OR prenatal OR perinatal OR parturition OR delivery, obstetric OR cesarean section OR labor OR Vacuum Extraction, Obstetrical OR Dystocia)
AND (fear OR anxiety OR "birth trauma" OR PTSD OR stress disorders)

LIMITS: language English, Swedish. Exclude Medline records

**Cochrane Library**((((fear OR anxiety OR "birth trauma" OR PTSD OR stress disorders))) AND ((birth OR childbirth OR "post partum" OR postpartum OR "post natal" OR postnatal OR puerperium OR antenatal OR prenatal OR perinatal OR parturition OR delivery, obstetric OR cesarean section OR Extraction, Obstetrical OR labor, induced)))

**PsycInfo**((ti(Childbirth OR Perinatal OR Postnatal OR Primipara OR Birth) AND ti(Anxiety OR Fear OR "birth trauma")) OR (ab(Childbirth OR Perinatal OR Postnatal OR Primipara OR Birth) AND ab(Anxiety OR Fear OR "birth trauma")) OR ((SU.EXACT("Labor (Childbirth)") OR SU.EXACT("Perinatal Period") OR SU.EXACT("Postnatal Period") OR SU.EXACT("Primipara") OR SU.EXACT("Birth")) AND (SU.EXACT("Anxiety") OR SU.EXACT("Birth Trauma") OR SU.EXACT("Fear"))) AND me.exact("Empirical Study" OR "Quantitative Study" OR "Longitudinal Study" OR "Interview" OR "Followup Study" OR "Literature Review" OR "Prospective Study" OR "Qualitative Study" OR "Clinical Case Study" OR "Treatment Outcome/Clinical Trial" OR "Retrospective Study" OR "Systematic Review" OR "Meta Analysis" OR "Focus Group")) AND la.exact("ENG")

**Scopus**
( ( ( TITLE-ABS-KEY ( birth )  OR  TITLE-ABS-KEY ( prenatal )  OR  TITLE-ABS-KEY ( perinatal )  OR  TITLE-ABS-KEY ( parturition )  OR  TITLE-ABS-KEY ( delivery,obstetric )  OR  TITLE-ABS-KEY ( cesarean  section )  OR  TITLE-ABS-KEY ( extraction,obstetrical )  OR  TITLE-ABS-KEY ( labor,induced )  OR  TITLE-ABS-KEY ( antenatal )  OR  TITLE-ABS-KEY ( puerperium )  OR  TITLE-ABS-KEY ( postnatal )  OR  TITLE-ABS-KEY ( "post natal" )  OR  TITLE-ABS-KEY ( postpartum )  OR  TITLE-ABS-KEY ( "post partum" )  OR  TITLE-ABS-KEY ( childbirth ) ) )  AND  ( TITLE-ABS-KEY ( fear )  OR  TITLE-ABS-KEY ( anxiety )  OR  TITLE-ABS-KEY ( "birth trauma" )  OR  TITLE-ABS-KEY ( ptsd )  OR  TITLE-ABS-KEY ( "stress disorders" ) ) )  AND  ( TITLE-ABS-KEY ( "Randomized Controlled Trial" )  OR  TITLE-ABS-KEY ( "Observational Study" )  OR  TITLE-ABS-KEY ( "Clinical Trial" )  OR  TITLE-ABS-KEY ( placebo* )  OR  TITLE-ABS-KEY ( random* )  OR  TITLE-ABS-KEY ( review )  OR  TITLE-ABS-KEY ( meta-analysis )  OR  TITLE-ABS-KEY ( questionnaire )  OR  TITLE-ABS-KEY ( interview )  OR  TITLE-ABS-KEY ( survey )  OR  TITLE-ABS-KEY ( cohort  study )  OR  TITLE-ABS-KEY ( focus  group ) )  AND  ( LIMIT-TO ( LANGUAGE ,  "English" )  OR  LIMIT-TO ( LANGUAGE ,  "Swedish" ) )
